# Supplementary figures and images for: HIF inhibitor topotecan has a neuroprotective effect in a murine retinal ischemia-reperfusion model
Source: PeerJ. 2019 Oct 4;7:e7849. doi: 10.7717/peerj.7849 (PMC6779112; doi:10.7717/peerj.7849)

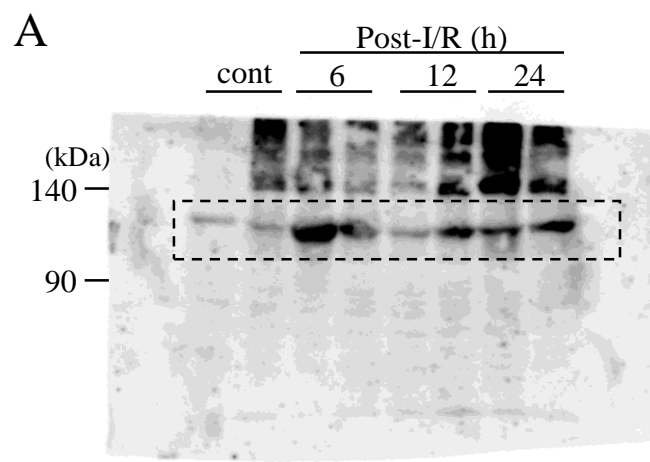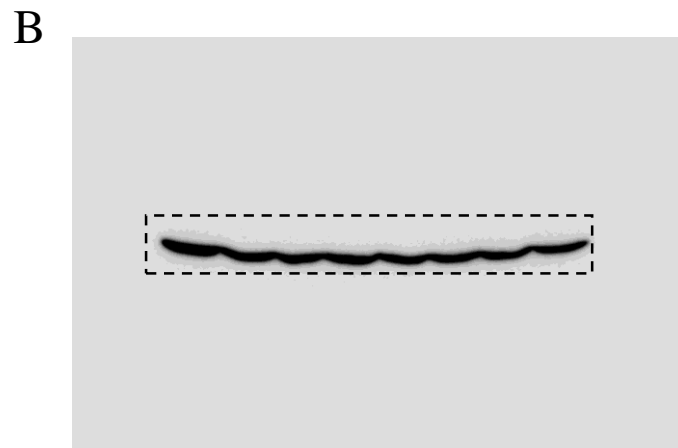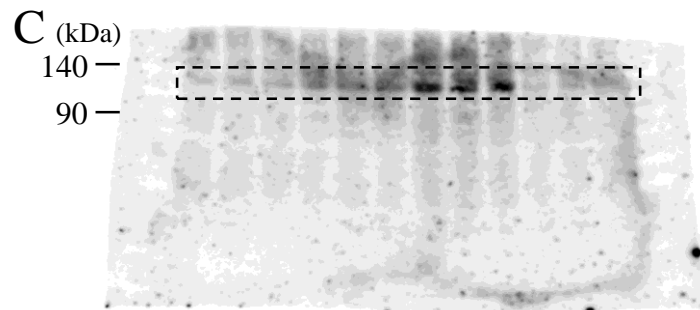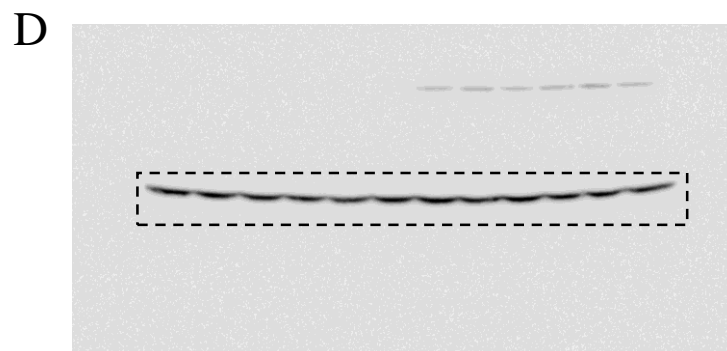

Supplement: Figure S1 [file peerj-07-7849-s001.pdf]
